# Antimeningococcal Vaccination Coverage Among Healthcare Workers in an Italian University Hospital

**DOI:** 10.3389/fpubh.2021.651100

**Published:** 2021-04-26

**Authors:** Vittorio Gattini, Marco Napoletano, Alessandra Bonotti, Aldo Mignani, Francesca Cosentino, Giovanni Guglielmi, Poupak Fallahi, Alfonso Cristaudo, Rudy Foddis

**Affiliations:** ^1^U. O. Medicina Preventiva del Lavoro, Azienda Ospedaliero-Universitaria Pisana, Pisa, Italy; ^2^School of Specialization in Occupational Medicine, University of Pisa, Pisa, Italy; ^3^Department of Translational Research and New Technologies in Medicine and Surgery, University of Pisa, Pisa, Italy

**Keywords:** antimeningococcal vaccine, vaccine coverage, healthcare worker, *Neisseria meningitidis*, *Neisseria meningitidis* vaccine

## Abstract

**Introduction:** Following an outbreak of meningococcal epidemic in 2015 and 2016 in Tuscany, we registered a higher demand for antimeningococcal vaccination (anti-Men ACWY) by Healthcare Workers of the University Hospital of Pisa [Azienda Ospedaliero Universitaria Pisana (AOUP)]. The purpose of this work is to analyze and discuss data on vaccination coverage resulting from this vaccination campaign.

**Materials and Methods:** We conducted a monocentric study about anti-Men vaccination in the healthcare workers of the AOUP following the outbreak of meningococcal meningitis that occurred mainly in the population of the Tuscan provinces of Pisa, Pistoia, Prato, and Florence. The variables under examination were age, sex, educational qualification, and job profile. Department healthcare workers were vaccinated with two types of conjugated tetravalent vaccines for the A, C, Y, and W135 strains. Data were analyzed using the SPSS software.

**Results:** The total population of the workers in AOUP was 7,188 subjects; the population considered in the study was 5,889. Between 2015 and 2017, a total of 2,423 subjects (41.1%) underwent anti-Men vaccination. Women, older HCWs, those with a lower educational qualification, doctors, and the HCWs of the maternal and child department, and imaging department recorded a statistically significant better vaccine compliance.

**Discussion:** The AOUP, implementing the program of the Tuscany Region of vaccination against *Neisseria meningitidis*, has contributed to reduce the incidence of invasive meningococcal disease. Some critical issues remain in the compliance of some sections of the population, despite the high level of adherence recorded in this case, probably also due to the great media coverage of the event.

## Highlights

- Great media coverage seems to increase compliance to anti-meningococcal vaccination;
- Healthcare workers dealing with more fragile patients tend to adhere more to vaccination even where there is no obligation;
- Vaccination coverage for *Neisseria meningitidis* in healthcare workers is still sub-optimal.

## Introduction

The invasive meningococcal disease (IMD) from *Neisseria meningitidis* is still a frequently fatal pathology. The bacterium currently counts 13 recognized serogroups, among which A, B, C, W135, and Y are the most frequently isolated in the case of illness (1).

Concerning adult population, anti-Men vaccination is recommended in subjects with an increased risk of contracting an invasive infection due to clinical conditions (e.g., subjects with hemoglobinopathies, functional or anatomic asplenia, and splenectomy candidates, those who suffer from congenital or acquired immunodepression, etc.) (2), or to socio-occupational status (e.g., military recruits, college students, and travelers to countries where meningococcal disease is hyperendemic or epidemic) (3). According to the World Health Organization (WHO), specifically for Healthcare Workers (HCWs), anti-Men vaccination is recommended for those employed in microbiology/bacteriological departments (deliberate biological risk or not). It is recommended also for HCW employed in other laboratories that are exposed to biological risks and deal with the execution of chemical–clinical, histo-, and pathological analysis on potentially infected biological materials (4).

In Italy, the anti-Men ACWY vaccination is not mandatory (not even recommended) for the HCWs, with the only exception of laboratory workers (2), who are at 65–184 times of higher risk to develop IMD than the general population (5). For laboratory staff, some European countries are licensing also anti-Men B vaccines (6).

The anti-Men ACWY is considered useful and appropriate for the HCWs employed in departments at high risk of transmission of airborne diseases.

IMD is an endemic disease accountable for an average mortality rate of 5–10% in developed nations, which, however, rises up to 20% if we consider cases complicated by sepsis (7). The ECDC “Annual epidemiological report for 2015” reported 3,112 cases of *Neisseria meningitidis* in Europe and an overall 0.6/100,000/year notification rate of IMD in EU countries, while in the previous year, there were 2,760 cases (8). Data on anti-Men vaccination coverage in the HCWs are limited in the international literature (9, 10) and, to date, we have not found any study concerning the topic carried out in Italy. It would therefore be important to have more data to establish the best strategies for promoting greater coverage in healthcare professionals. The purpose of this work is to analyze and discuss the data of a single center related to the vaccination coverage resulting from this campaign and to compare it with those present at the time in the literature.

## Materials and Methods

In the period 2015–2017, the anti-Men vaccination with Menveo (GlaxoSmithKline Vaccines S.r.l., Brentford, UK) or Nimenrix (Pfizer Inc, Sandwitch, UK)-conjugated tetravalent vaccine was offered free of charge to all employees (*n* = 7,188) of the Azienda Ospedaliero-Universitaria Pisana (AOUP). The exclusion criteria adopted were age > 75 years and the administrative task because this job profile is not related to an interaction with potential infected patients. After the application of exclusion criteria, the studied subjects were 5,889 (1,294 were excluded for the administrative job profile and five for the age > 75 years). Vaccinations were administered by the staff of the Preventive and Occupational Medicine Department of the AOUP within a 3-year period. The anti-Men vaccine was offered at the time of the medical examination for occupational purposes. At the time of vaccination, personal and working data were collected (if not yet present in our database), and both recorded in the management software of the health risk file (Asped2000NE). Recorded data were sociodemographic variables of HCWs, such as age, sex, educational qualification, job profile, ward, and department. Each subject gave his oral and written consent to vaccination protocol and to enrollment in the research, approved by the local ethical committee.

The statistical analysis was performed using SPSS v 20.0 (Statistical Package for Social Sciences). All categorical data (sex, education, job profile, department, and operative unit) were expressed by their frequencies (number and percentage), whereas age was analyzed as mean, standard deviation, and minimum and maximum values. The Geriatric, Oncology, Hematology, Pediatric Oncohematology, Radiotherapy, Infectious Diseases, and Burn Center operative units were considered as a single group in order to verify the prevalence in those working with patients at higher risk of immunodeficiency vs. the other HCWs, in comparable-sized samples.

The statistical differences between several groups were assessed using the Chi-Square test and *T*-Student test. The statistical significance was accepted for *p* < 0.05.

## Results

The studied population, enrolled from January 1, 2015 to December 31, 2017, consisted of 5,889 subjects in health care surveillance for their occupational risk factors, with a mean age of 44.91 years (SD = 11.17; min = 23 and max = 75), of 44.5 ± 11.5 years in the unvaccinated group, and of 45.5 ± 10.7 in the vaccine group. Of the population, 68.1% (*n* = 4,010) were females and 31.9% were males (*n* = 1,879). Considering all departments, 2,423 subjects (41.1%) accepted the anti-Men vaccination. The vaccination was administered to 666 workers in 2015 (24.5%), to 1,310 in 2016 (54.1%), and to 447 workers in 2017 (18.4%). In Table 1, the demographic and occupational data were reported about unvaccinated and vaccinated subjects. Concerning the job profile, we have grouped into the “Technicians” category all non-medical health professions such as, for example, biomedical laboratory technicians, radiology technicians, physiotherapists, rehabilitation technicians, logotherapists, etc.

**Table 1:** Demographic and occupational data.

**Tot (*n* = 5,889)**	**Unvaccinated**	**Vaccinated**	***P*-value**
	**(*n* = 3,466)**	**(*n* = 2,423)**	
**Age (years)**			
Mean, SD	44.5 ± 11.5	45.5 ± 10.7	<0.0001
**Sex (*****n*****)**			
Male (1,879)	1,166 (62.1%)	713 (37.9%)	<0.0001
Female (4,010)	2,300 (57.4%)	1,710 (42.6%)	
**Education (*****n*****)**			
Secondary school (379)	119 (31.4%)	260 (68.6%)	<0.0001
High school (1,582)	981 (62,0%)	601 (38.0%)	
University (3,928)	2,366 (60.2%)	1,562 (39.8%)	
**Job profile (*****n*****)**			
Technicians (898)	593 (66.0%)	305 (34%)	<0.0001
Nurses (2,715)	1,586 (58.4%)	1,129 (41.6%)	
Doctors (2,276)	1,287 (56.5%)	989 (43.5%)	

In Table 1, *p*-value concerns the comparison between sex, education, and different job profiles in the vaccine group.

**Figure 1:**
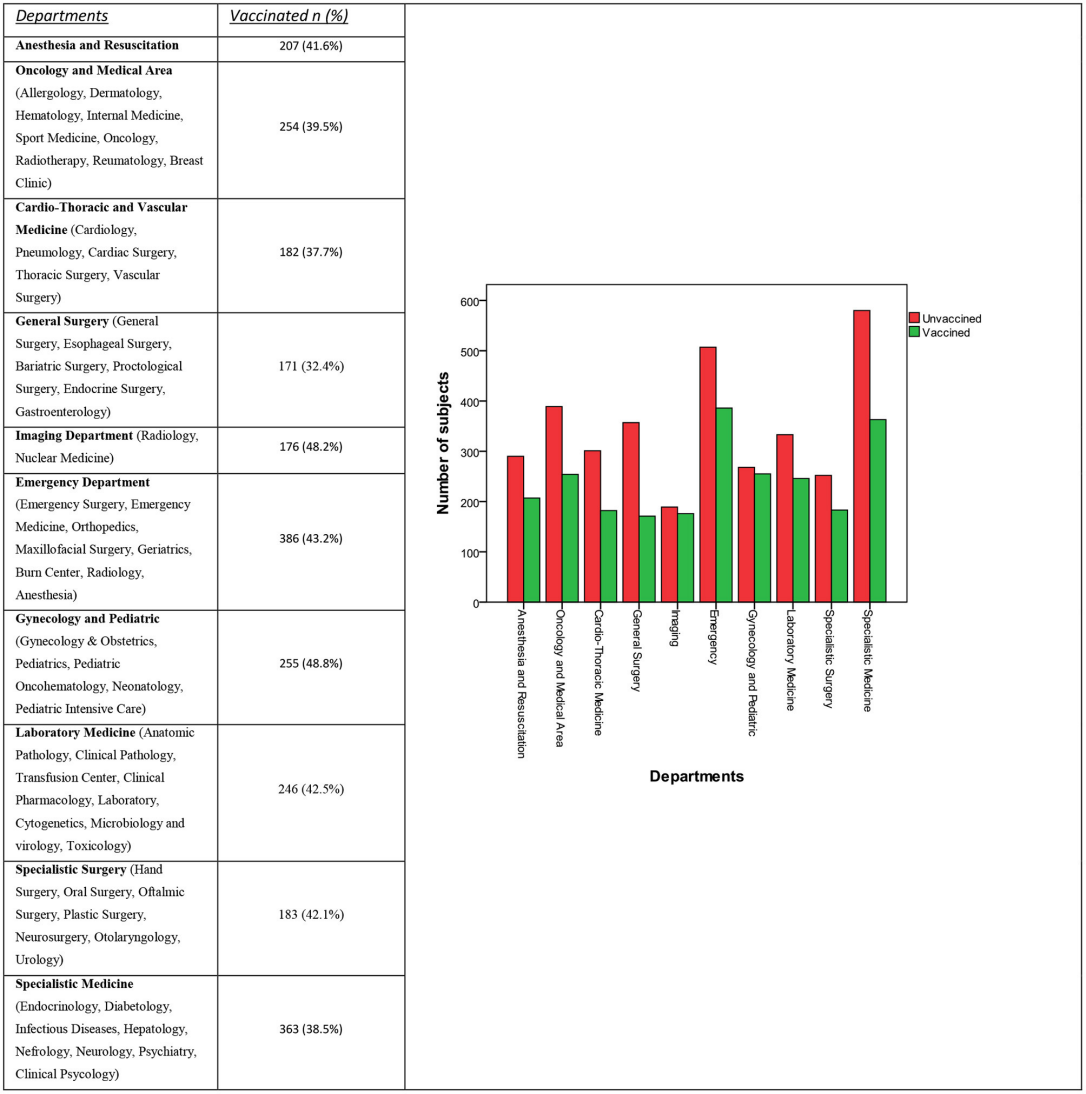
Distribution of vaccine to unvaccinated subjects in different departments, in number and percentage.

All the operative units of AOUP were included in the different departments, based on their operative field, and a graphic representation of unvaccinated and vaccinated subjects is reported in Figure 1.

The average prevalence of anti-Men vaccination within the entire population of AOUP HCWs was 41.1%. The anti-Men vaccination rates among workers from both the Gynecology/Pediatric and the Imaging Department were significantly higher (*p* < 0.0001 and *p* = 0.003, respectively) than the average rate of all the other HCWs in AOUP (40.4, 40.7%). We also found that some operative units (i.e., Geriatric, Oncology, Hematology, Pediatric Oncohematology, Radiotherapy, Infectious Diseases, and Burn Center operative units) showed singularly as well as cumulatively (44.3 vs. 40.8%, *p* = 0.081) a higher, though not statically significant, rate of anti-Men vaccination when compared with the rest of the AOUP HCWs.

## Discussion

The AOUP is one of the largest hospitals in Italy, with high specialization in several clinical fields. In 2017, a total of 58,247 hospitalizations were performed, of which 46,964 were ordinary hospitalizations and 11,283 day hospital admissions, 32,142 for surgical and 26,105 medical treatment (11).

Since the great number of patients is afferent to our hospital, it is important that HCWs are covered from most dangerous infective diseases, like measles, rubella, chickenpox, and meningitis (12).

In Italy, the incidence of IMD in the 3-year period between January 2015 and December 2017 was of 0.31 cases/100,000 inhabitants in 2015 and 0.38 cases/100,000 inhabitants in 2016, lower than the European average of 0.6 cases/100,000 inhabitants (in 2015, the most recent data available)^1^. From 2015, in Tuscany, an anomalous increase in IMD cases from meningococcus was observed, compared with the previous years and the average national data. This situation lasted until 2016: the cases recorded of meningococcal disease in this 2-year period were as many as 61 with an incidence of 0.81/100,000 inhabitants (compared with the 22 total cases notified in the years 2007–2014 with an incidence average of 0.2/100,000 inhabitants). It provoked the death of 13 people, with a lethality of 21.3%. In 2017, thanks also to the undertaken extraordinary vaccination campaign, there was a significant overall reduction: only nine cases occurred, without any death, with an incidence rate falling down to previous statistics (0.24/100,000 inhabitants). The most frequently isolated serogroup was C, followed by the serogroup B, and finally the serogroup Y^1^.

Following the outbreak of IMD in Tuscany in 2015, there has been great media coverage of every case that could resemble meningitis, and this highly contributed to a collective psychosis around that. So there has been great debate about the need to propose the anti-Men vaccination at least to the categories with utmost risk of contracting the infection.

The Tuscany Region offered free vaccination from 2015 to December 31, 2018 to people of age between 20 and 45 years living in Tuscany, who got in touch with a patient that further developed IMD or to those who attended at least 10 days earlier the same places or communities where a case of IMD happened^2^.

In Italian legislation, there is no vaccination requirement regarding *N. meningitides* (2). Considering, however, that HCWs have an additional risk of contracting this infection compared with the general population, as part of the catch-up campaign, the AOUP, the Preventive and Occupational Medicine Department proposed free vaccination against *N. meningitidis* A, C, W125, and Y to all HCWs undergoing health care surveillance (*n* = 5,889), excluding administrative workers.

In this study, workers with a major attitude to be vaccinated vs. *N. meningitidis* were females (42.6%, *p* < 0.0001), were older (45.5 ± 10.7 vs. 44.5 ± 11.5, *p* < 0.0001), attended secondary schools (68.6%, *p* < 0.0001), and considering job titles, physicians (43.5%, *p* < 0.0001). These data are in agreement with what were reported by the few previous studies (5, 6). The greater aptitude for vaccination of workers with a lower level of education may be explained by the greater susceptibility to the massive media campaign undertaken by the media during the outbreak period. Another factor to be considered is that, this portion of the population represents the largest pool of previously unvaccinated individuals and, therefore, those who, more than others, adhere to free vaccination campaigns.

If we consider the temporal distribution of the administration of the anti-Men vaccine, we can see how the peak of requests occurred in 2016 (54.1%). This can be easily explained by the peak in the number of cases of IMD by *N. meningitidis* recorded in that year, and by the fact that in the following period, the media attention toward the outbreak has been reduced also due to the decrease in the incidence of pathology.

From the analysis of the vaccination coverage within the various departments, it is not surprising that the greatest prevalence is found in the maternal and child department (48.8 vs. 40.4%, *p* < 0.0001). Here, in fact, HCWs are more sensitive to the problem of being able to transmit an infectious disease to a population particularly at risk of major complications such as pediatric patients.

Another department that showed an increased prevalence of vaccination against *N. meningitidis* was the Imaging Department (48.2 vs. 40.7%, *p* = 0.003). In this case, the largest proportion of employees who may have raised the average may report to the Imaging Department of the Emergency Department, typically more in contact with patients at risk of transmitting infectious diseases.

Two other departments that showed a high adherence to the vaccination campaign offered by the AOUP were the Emergency department and the “fragile patients” department, in which we artificially collected the Geriatric, Oncology, Hematology, Pediatric Oncohematology, Radiotherapy, Infectious Diseases, and Burn Center operative units in function of the greater presence of patients particularly at risk of acquiring infections or of developing major complications in case of infection. In these departments, however, a statistically significant difference comparing with the remaining AOUP working population was not highlighted, but only an increasing trend. We can explain these results considering that in these departments, workers were generally already vaccinated against the most dangerous pathogens, including *N. meningitidis*.

A limitation of our study is that the vaccine adherence data in some departments may have been underestimated. This may have happened because we did not know the HCW vaccination coverage data before the campaign was implemented by our facility. Another limitation of our study is that our results were derived from a temporally and geographically circumscribed observation. Therefore, the replication of our research through a multicenter design, over a longer time frame, and a larger population of healthcare professionals is needed.

In the considered population, we found a statistically significant difference in the acceptance of the anti-Men vaccine on the basis of the work profile, with a greater compliance by the medical staff with respect to the nursing and technical ones (*p* < 0.0001). These data are in partial disagreement with what were reported in literature, in particular, from the study of Madani et al. (10), which did not find any statistically significant difference in the vaccination compliance between doctors and nurses. Other studies did not investigate the compliance in vaccination based on the job profile.

On the contrary, according to what was reported by Madani et al. (10), also in our population, we found a statistically significant difference in the acceptance of vaccination based on the level of education. As in the aforementioned study, there was greater compliance by the subjects with a lower level of education than those with higher educational qualifications. Subjects with a lower level of education historically represent a population that is more reluctant to vaccination, especially in Italy, where the disinformation linked to possible adverse events to vaccines is always greater. Therefore, the data detected in our study can be explained, at least in part, by the fact that this population represents the largest reservoir of unvaccinated subjects and, therefore, the largest slice of the population to which vaccination will be destined. Moreover, these subjects could be more sensitive to the media campaign undertaken by the local and national media to cover the Tuscan meningitis outbreak.

These data show how the intervention of the AOUP in proposing free anti-Men vaccination to the HCWs, in association with the program of the Tuscan Region, managed to reduce the cases of meningitis up to realign the data of the incidence with that of the other Italian regions.

## Data Availability Statement

The original contributions presented in the study are included in the article/supplementary material, further inquiries can be directed to the corresponding author/s.

## Ethics Statement

Ethical review and approval was not required for the study on human participants in accordance with the local legislation and institutional requirements. Written informed consent for participation was not required for this study in accordance with the national legislation and the institutional requirements.

## Author Contributions

VG: conceptualization, methodology, supervision, validation, and writing–review and editing. MN: conceptualization, investigation, and writing–original draft. AB: data curation, formal analysis, and software. AM, FC, and GG: project administration and validation. PF and RF: project administration, supervision, validation, and writing–review and editing. AC: project administration, resources, supervision, software, validation, and writing–review and editing. All authors contributed to the article and approved the submitted version.

## Conflict of Interest

The authors declare that the research was conducted in the absence of any commercial or financial relationships that could be construed as a potential conflict of interest.

## Footnotes

^1^Italian National Health Institute. Surveillance data on invasive bacterial diseases updated on 19 March 2018.

^2^Delibera Regione Toscana n.85 del 16-02-2016.
